# Cluster analysis of phenotypes of patients with Behçet’s syndrome: a large cohort study from a referral center in China

**DOI:** 10.1186/s13075-021-02429-7

**Published:** 2021-01-30

**Authors:** Jun Zou, Jian-feng Luo, Yan Shen, Jian-fei Cai, Jian-long Guan

**Affiliations:** 1 Division of Rheumatology and Immunology, Shanghai Key Laboratory of Clinical Geriatric Medicine, Research Center on Aging and Medicine, Huadong Hospital, Fudan University, 200040 Shanghai, People’s Republic of China; 2 Department of Biostatistics, School of Public Health, Fudan University, Shanghai, People’s Republic of China

**Keywords:** Behçet’s syndrome, Phenotype, Sex, Cluster analysis, Clinical manifestation, Major organ involvement

## Abstract

**Introduction:**

Behcet’s syndrome (BS) is a complex, heterogeneous disorder. However, classification of its subgroups is still debated. The purpose of this study was to investigate the clinical features and aggregation of patients with BS in China, based on manifestations and organ involvements.

**Methods:**

This was a cross-sectional study of BS patients in Huadong Hospital of Fudan University between September 2012 and January 2020. We calculated relative risks (RRs) of clinical variables according to sex. Moreover, we conducted a hierarchical cluster analysis applied according to eighteen variables to determine subgroups of patients.

**Results:**

A total of 860 BS patients were included. Male sex was associated with ocular involvement (RR 2.32, 95% CI 1.67, 3.22, *P* < 0.0001), vascular involvement (RR 2.00, 95% CI 1.23, 3.23, *P* = 0.004), cardiac lesion (RR 5.46, 95% CI 2.33, 12.77, *P* < 0.0001), and central nervous system involvement (RR 2.95, 95% CI 1.07, 6.78, *P* = 0.007) and was negatively associated with genital ulcers (RR 0.84, 95% CI 0.79, 0.91, *P* < 0.0001). Five clusters (C1–C5) were observed. C1 (*n* = 307) showed the skin and mucosa type. In C2 (*n* = 124), all had articular involvement, barely having major organ involvement except for 18 cases with intestinal lesions. In C3 (*n* = 156), the gastrointestinal type, 144 patients presented with intestinal involvement, and 36 patients with esophageal ulcers. In C4 (*n* = 142), all subjects presented with uveitis. C5 (*n* = 131) consisted of 44 patients with cardiac lesions, 58 with vascular involvement, and 26 cases having central nervous system involvement.

**Conclusion:**

Our analysis confirmed sex differences in phenotypes of BS. Cluster analysis identified gastrointestinal, uveitis, and cardiovascular involvement cluster separately in different subsets, which represents the most commonly involved organs. Further research is required to replicate and clarify the patterns of phenotype in BS.

## Significance and innovations

- Our study confirmed sex-related phenotypes, especially the association between male sex and cardiac, arterial disease.
- Five subgroups were identified by cluster analysis, with gastrointestinal, uveitis, and cardiovascular and CNS clusters representing commonly involved organs.

Behçet’s syndrome (BS) is a rare disorder that causes various blood vessel inflammations with a unique geographic distribution and obscure etiology [1]. In the 1930s, it was successively described by Benedict Adamantiades and Hulusi Behçet as a triad of aphthous oral ulcers, genital lesions, and hypopyon [2]. In honor of the contribution of both scientists, it is also named as *Adamantiades-Behçet’s disease (ABD)*. Thereafter, major organ involvements in patients with BS have been reported, such as neurological [3], cardiovascular [4, 5], and intestinal manifestations [6]. The “atypical” manifestations of BS represent the heterogeneous characteristics of the disorder [7]. Although BS affects nearly every organ, usually few organs are involved in the same patient [8]. A recent meta-analysis using pooled BS cases of 2061–13,995 estimated [9] that the frequencies of 16 common disease-related manifestations were below 50%, except for skin-mucosa lesions, which indicates a variety of combinations of major organ involvements among individual patients. The symptoms and major organ involvement of BS tend to vary among sex, age [9–11], and ethnic groups [12–14]. Epidemiological studies on BS are always important because the diagnosis of BS is clinical, and changes in clinical characteristics and severity may be observed during the course of the disorder [14]. Accurate definition of phenotypic clusters is of crucial importance for proper management. There are some phenotype studies mainly using factor analysis [15], correspondence analysis [16], or logistic regression analysis [17] to explore patterns of organ associations from different countries. Recently, Seyahi [18] reviewed and proposed six phenotypes: skin-mucosa involvement, joint involvement, vascular involvement, eye involvement, parenchymal neurological involvement, and gastrointestinal involvement.

China is an endemic area of BS. Nevertheless, epidemiological researches in China are inadequate and limited by either enrolling a small number of subjects [19–21] or focusing on one specific subgroup of BS [22–24]. We could not get a panoramic view of clinical phenotypes of BS in China from the limited data previously published. There is great interest in better characterizing the heterogeneity in this complex disease [25]. Disease phenotypes defining clinical subgroups could offer us a chance to decipher the pathogenesis and hence provide precision medicine [26].

Therefore, the purpose of the present study is to identify sex-associated differences in manifestations and major organ involvements. In order to minimize subjective bias, we employ an unsupervised clustering analysis to define certain clinical subsets with homogeneous phenotype and clinical manifestations.

## Patients and methods

### Cohort overview

A cross-sectional study of BS patients was conducted in the Department of Rheumatology and Immunology in Huadong Hospital, Fudan University, from September 2012 to January 2020. The revision of International Study Group criteria (ISG) [27], Japan revised [28], Cheng and Zhang criteria (China) [29], and International Criteria for BS (ICBD) [30] were selected for inclusion. We included patients who satisfied at least one of the four selected classification criteria. The final diagnosis was verified by at least 2 rheumatologists. Detailed clinical and laboratory data were recorded, including demographic data, laboratory assessments, imaging studies, and pathological findings. This study was approved by the ethics committee of Huadong Hospital and all patients gave consent to participate in the study.

Previously, we found BS patients could concurrently associate with myelodysplastic syndrome (MDS) [31]. Accordingly, exclusion criteria included malignancies (except for MDS), infectious diseases, or other inflammatory rheumatic disorders.

### Assessment of clinical manifestation, major organ involvement, and severity

Organ involvement was assessed by reviewing the patient’s symptoms, past medical history, physical examination, laboratory studies, imaging examinations, and endoscopy findings. Ophthalmologic data recorded the type of uveitis (namely, anterior, posterior, or panuveitis), laterality, ocular findings, and ocular complications [32]. Diagnosis of intestinal BS was confirmed with extraintestinal systemic manifestations, and the characteristic endoscopic, histopathologic, and radiological features, which helped to distinguish intestinal BD from Crohn’s disease [33]. The classification of major vascular involvements in BS was adopted [34]. Vascular involvement was defined as deep venous thrombosis, major vein (vena cava, hepatic) thrombosis, and arterial thrombosis or aneurysms, which were detected by Doppler ultrasonography and (or) magnetic resonance imaging (MRI) and (or) computerized tomography (CT) [35]. Cardiac lesions were documented as valvular regurgitation, intracardiac thrombi [36], and coronary artery disease [24], which were documented by echocardiography or coronary angiography and (or) CT. Atherosclerosis or other causes of cardiac lesions were carefully excluded. MDS was diagnosed and classified according to WHO classification [37], while patients had typical BS manifestations. Central nervous system (CNS) included inflammatory parenchymal lesions, and extra-parenchymal forms causing cerebral venous sinus thrombosis [38].

Disease severity was assessed by Krause’s score [13].

### Statistical analysis

The software program SPSS (v. 20, Chicago, IL) was used for statistical analyses. Values are expressed as means ± SD or medians with 25–75% ranges, whichever was appropriate depending on whether the data were normally distributed. Student’s test or the Mann-Whitney*U* test was used to compare numerical variables between groups. The chi-square or Fisher’s exact test was used to compare categorical variables. *P* values < 0.05 were considered statistically significant.

The TwoStep Cluster Analysis began with the selection of variables, which were classified as continuous or categorical. Continuous variables were age, age at onset, duration of disease, and Krause score. Categorical variables were sex, clinical manifestation (recurrent oral ulcers, genital ulcers, erythema nodosum, papulopustular lesions, joint involvement), and major organ involvement (uveitis, gastrointestinal involvement, cardiovascular involvement, parenchymal involvement, cerebral venous sinus thrombosis (CVST), cerebral arterial involvement, and MDS). In total, 18 variables were included for cluster analysis apart from the classification criteria; the rest of the variables are shown in Table 1. The log-likelihood method was used to determine inter-subject distance and specific classification of participants.

**Table 1:** Relative risks by gender for clinical variables among patients with Behçet’s syndrome

Variable	Total (*n* = 860)	Men (*n* = 462)	Women (*n* = 398)	RR (male: female) (95% CI)	*P* value
Oral ulcer	856 (99.5)	459 (99.4)	397 (99.7)	1.00 (0.99, 1.00)	*P* = 0.628
Genital ulcer	663 (77.1)	328 (71.0)	335 (84.2)	0.84 (0.79, 0.91)	*P* < 0.0001
Skin lesions	477 (55.5)	273 (59.1)	204 (51.3)	1.15 (1.02, 1.30)	*P* = 0.021
Erythema nodosum	344 (40.0)	183 (39.6)	161 (40.5)	0.98 (0.83, 1.15)	*P* = 0.802
Folliculitis	208 (24.2)	138 (29.9)	70 (17.6)	1.70 (1.32, 2.19)	*P* < 0.0001
Arthritis	104 (12.1)	59 (12.8)	45 (11.3)	1.13 (0.79, 1.63)	*P* = 0.511
Arthralgia	77 (9.0)	46 (10.0)	31 (7.8)	1.28 (0.83, 1.98)	*P* = 0.267
Ocular involvement	155 (18.0)	113 (24.5)	42 (10.6)	2.32 (1.67, 3.22)	*P* < 0.0001
Anterior uveitis	18 (2.1)	9 (1.9)	9 (2.3)	0.86 (0.35, 2.15)	*P* = 0.749
Panuveitis	137 (15.9)	104 (22.5)	33 (8.3)	2.72 (1.88, 3.92)	P < 0.0001
Intestinal involvement	178 (20.7)	92 (19.9)	86 (21.6)	0.92 (0.71, 1.20)	*P* = 0.541
Intestinal erosion	7 (0.8)	3 (0.6)	4 (1.0)	0.65 (0.15, 2.87)	*P* = 0.710
Intestinal ulcer	141 (16.4)	71 (15.4)	70 (17.6)	0.87 (0.65, 1.18)	*P* = 0.381
Intestinal perforation	30 (3.5)	18 (3.9)	12 (3.0)	1.29 (0.63, 2.65)	*P* = 0.483
Esophageal ulcer	36 (4.2)	21 (4.5)	15 (3.8)	1.21 (0.63, 2.31)	*P* = 0.571
Vascular involvement	73 (8.5)	51 (11.0)	22 (5.5)	2.00 (1.23, 3.23)	*P* = 0.004
Deep venous thrombosis	29 (3.4)	22 (4.8)	7 (1.8)	2.71 (1.17, 6.27)	*P* = 0.015
Major vein (vena cava, hepatic) thrombosis	7 (0.8)	6 (1.3)	1 (0.3)	5.17 (0.63, 42.75)	*P* = 0.131
Arterial thrombosis or aneurysms	46 (5.3)	32 (6.9)	14 (3.5)	1.97 (1.07, 3.64)	*P* = 0.027
Cardiac lesion	44 (5.1)	38 (8.2)	6 (1.5)	5.46 (2.33, 12.77)	*P* < 0.0001
CNS disorder	31 (3.6)	24 (5.2)	7 (1.8)	2.95 (1.29, 6.78)	*P* = 0.007
Parenchymal involvements	23 (2.7)	16 (3.5)	7 (1.8)	1.97 (0.82, 4.74)	*P* = 0.122
MDS	6 (0.7)	1 (0.2)	5 (1.3)	0.17 (0.02, 1.47)	*P* = 0.101

Values presented as *n* (%) unless otherwise stated. *CNS* central nervous system, *MDS* myelodysplastic syndrome

## Results

### Demographics, manifestations, and major organ involvements

We enrolled 860 patients with BS. Among them, 424, 838, 759, and 427 cases satisfied Japan revised, Cheng and Zhang criteria (China), ICBD criteria, and ISG criteria, respectively.

The median age of patients was 36 years (interquartile range, IQR 28–47 years). The median age at onset was 27 years (IQR 20–36 years) and the median disease duration was 7 years (IQR 3–10 years). The median Krause score was 4 (IQR 3–5). The sex ratio in our cohort was M:F = 1.16:1. Oral aphthous ulcers were the most common manifestation; the prevalence of oral ulcers was 99.5%. The frequencies of other mucocutaneous and joint manifestations were genital ulcers 77.1%, erythema nodosum 40.0%, papulopustular lesions 24.2%, and arthritis or arthralgia 21.0%. Intestinal involvements were the most common major organ involvements in our cohort with a prevalence of 20.7%, followed by uveitis with 18.0%; vascular involvement 8.5%, including 29 cases with deep venous thrombosis, 7 with major vein (vena cava, hepatic) thrombosis, and 46 with arterial involvement; cardiac lesion 5.1% (valvular heart complications in 38 cases, ventricular intracardiac thrombus in 2, coronary involvement in 3, and myocarditis in 1 case); esophageal ulcer 4.2%; CNS involvement 3.6% (19 patients with parenchymal lesions, two having cerebral arterial involvement, cerebral venous sinus thrombosis in four cases, four with parenchymal lesions and brain aneurysm, and two having meningitis); and MDS 0.7%. The detailed demographic and clinical characteristics are shown in Table 1.

### Sex-phenotype analysis

In regard to sex-associated clinical features (Table 1), male sex was correlated with ocular involvement [RR (male: female) 2.32 (95% CI 1.67, 3.22)], including panuveitis [2.72 (1.88, 3.92)]; vascular involvement [2.00 (1.23, 3.23)], including deep venous thrombosis [2.71 (1.17, 6.27)] and arterial thrombosis or aneurysms [1.97 (1.07, 3.64)]; cardiac lesions [5.46 (2.33, 12.77)]; and central nervous system involvement [2.73 (1.10, 6.76)]. Male patients were more likely to have parenchymal lesions (16/462) as compared with female (7/398), but without statistical significance (*P* = 0.141). In addition, male sex was associated with skin manifestation [1.15 (1.02, 1.30)] and papulopustular lesions [1.70 (1.32, 2.19)]. Female sex was associated with genital ulcers [RR (male to female) 0.84 (0.79, 0.91)].

Noticeably, no sex difference was found in anterior uveitis, intestinal lesions (including intestinal erosion, ulcer and perforation), and esophageal ulcers.

We previously observed that the incidence of ocular involvement was lower among our gastrointestinal Behçet’s syndrome (GIBS) patients than among those with BS without GI lesions (0% vs 28%) [39]. Thus, we analyzed the association between ocular disease and intestinal involvement. We found that intestinal involvement was negatively associated with uveitis [0.26, (0.14, 0.49), *P* < 0.0001].

### Cluster analysis

Five clusters were generated with distinct features. The characteristics of each cluster are listed in Table 2.

**Table 2:** Characteristics of patients with Behçet’s syndrome after clustering on clinical manifestations

Characteristics	C1 (*n* = 307)	C2 (*n* = 124)	C3 (*n* = 156)	C4 (*n* = 142)	C5 (*n* = 131)
**Demographics**					
Age (IQR), years	36 (27–47)	39 (29–48)	37 (28–48)	33 (27–42)	37 (29–44)
Age at onset (IQR), years	28 (20–38)	28 (19–37)	28 (19–38)	25 (20–32)	27 (21–35)
Disease duration (IQR), years	6 (3–10)	9 (4–10)	6 (3–10)	7 (4–10)	6 (3–10)
Krause score	3 (2–3)	4 (3.3–5)	4 (4–5)	6 (5–6)	6 (5–7)
Sex ratio (M/F)	0.64:1	0.97:1	1.08:1	2.94:1	2.54:1
**Classification criteria**					
JPN (%)	52.4	41.9	22.4	83.8	43.5
CHN (%)	100	99.2	94.2	99.3	91.6
ICBD (%)	100	79.0	62.8	99.3	87.8
ISG (%)	52.8	45.2	23.1	81.7	43.5
**Clinical manifestation**					
Oral ulceration (%)	100	100	98.0	99.2	100
Genital ulceration (%)	100	75.8	61.5	58.5	63.4
Erythema nodosum (%)	40.7	44.4	19.2	48.6	49.6
Folliculitis (%)	20.8	28.2	25.6	28.2	22.1
Arthritis/arthralgia (%)	0	100	1.3	16.9	23.7
**Major organ involvement**					
Uveitis (%)	0	0	0	100	9.9
Intestinal involvement (%)	0	14.5	92.3	5.6	6.1
Esophageal lesions (%)	0	1.6	20.5	0.7	0.8
Vascular involvement (%)	0	0	0	0	44.3
Cardiac lesion (%)	0	0	0	0	33.6
Myelodysplastic syndrome (%)	0.3	1.6	1.9	0	0
Central nervous system involvement (%)	0	0.8	1.9	0.7	19.8

Values presented as *n* (%) unless otherwise stated. *JPN* Diagnostic criteria of the Behçet’s disease research committee of Japan (1987 revision), *CHN* the Chinese version of Behçet’s disease criteria created by Cheng and Zhang, *ICBD* International Criteria for Behçet’s disease, *ISG* International Study Group for Behçet’s disease

#### The first cluster (C1, *n* = 307, 35.7%)—skin and mucosa type, late-onset, female dominance

This was the largest group; it consisted of subjects with a median age at onset of 28 years (IQR 20–38 years), female predominant cluster sex ratio (male to female) = 0.64:1. All patients had genital ulcers. The prevalence of erythema nodosum was 40.7% and that of papulopustular lesions was 20.8%. This group had no major organ involvement, except for MDS in one case. The proportion of subjects meeting Japan revised, Cheng and Zhang, ICBD, and ISG criteria was respectively 52.4%, 100%, 100%, and 52.8%. Disease severity was low, the median Krause score = 3.

#### C2 (*n* = 124, 14.4%)—joint involvement type, late-onset, sex ratio (male to female, 0.97:1)

The subjects in C2 had a median age at onset of 28 years (IQR 19–37 years). All had joint involvement (arthritis or arthralgia). Papulopustular lesions presented in 28.2% cases. Major organ involvement was rarely seen, except for 18 cases of intestinal involvement and 2 cases of esophageal lesions gathered in this cluster. Patients met ICBD (79.0%) and Cheng and Zhang criteria (99.2%), while 41.9% satisfied Japan revised criteria and 45.2% ISG criteria. The median Krause Score was 4.

#### C3 (*n* = 156, 18.1%)—GIBS type, late-onset, sex ratio (male to female, 1.08:1)

The majority had intestinal lesions (92.3%), including intestinal erosion (5 cases), ulcers (117 cases) and perforation (22 cases), and had a median age at onset of 28 years (IQR 19–38 years). Most patients had oral ulcers (98.0%), genital ulceration (61.5%), and papulopustular lesions (25.6%), while the rate of erythematous nodosum (19.2%) was relatively lower than in the other clusters. No patients presented with ocular lesions or cardiovascular involvement. Esophageal ulceration was present in 32 cases of C3. Intestinal involvement was strongly related to esophageal ulcers [RR 6.02, (3.15, 11.53), *P* < 0.0001]. Three subjects had MDS in C3. Patients of BS associated with MDS had a higher risk of intestinal lesion than those without [RR 4.16, (2.31, 7.47), *P* = 0.008].

The proportion of subjects meeting the four classification criteria (JPN, CHN, ICBD, ISG) was 22.4%, 94.2%, 62.8%, and 23.1% respectively. The median Krause score was 4.

#### C4 (*n* = 142, 16.5%)—uveitis type, young male (male to female ratio, 2.94:1)

All subjects had ocular lesions, including anterior uveitis (16 cases) and panuveitis (126 cases), and the median age at onset was 25 years (IQR 20–32 years). Oral aphthous ulcers were seen in 99.2%, genital ulcers in 58.5%, and the frequency of skin manifestations was similar to that in C2. Inversely, patients in this group rarely had intestinal involvement (5.6%), no vascular or cardiac lesions. 83.8%, 99.3%, 99.3%, and 81.7% subjects satisfied JPN, CHN, ICBD, and ISG criteria, respectively. The median Krause score was 6.

#### C5 (*n* = 131, 15.2%)—cardiovascular type, male (male to female ratio, 2.54:1)

**Fig. 1:**
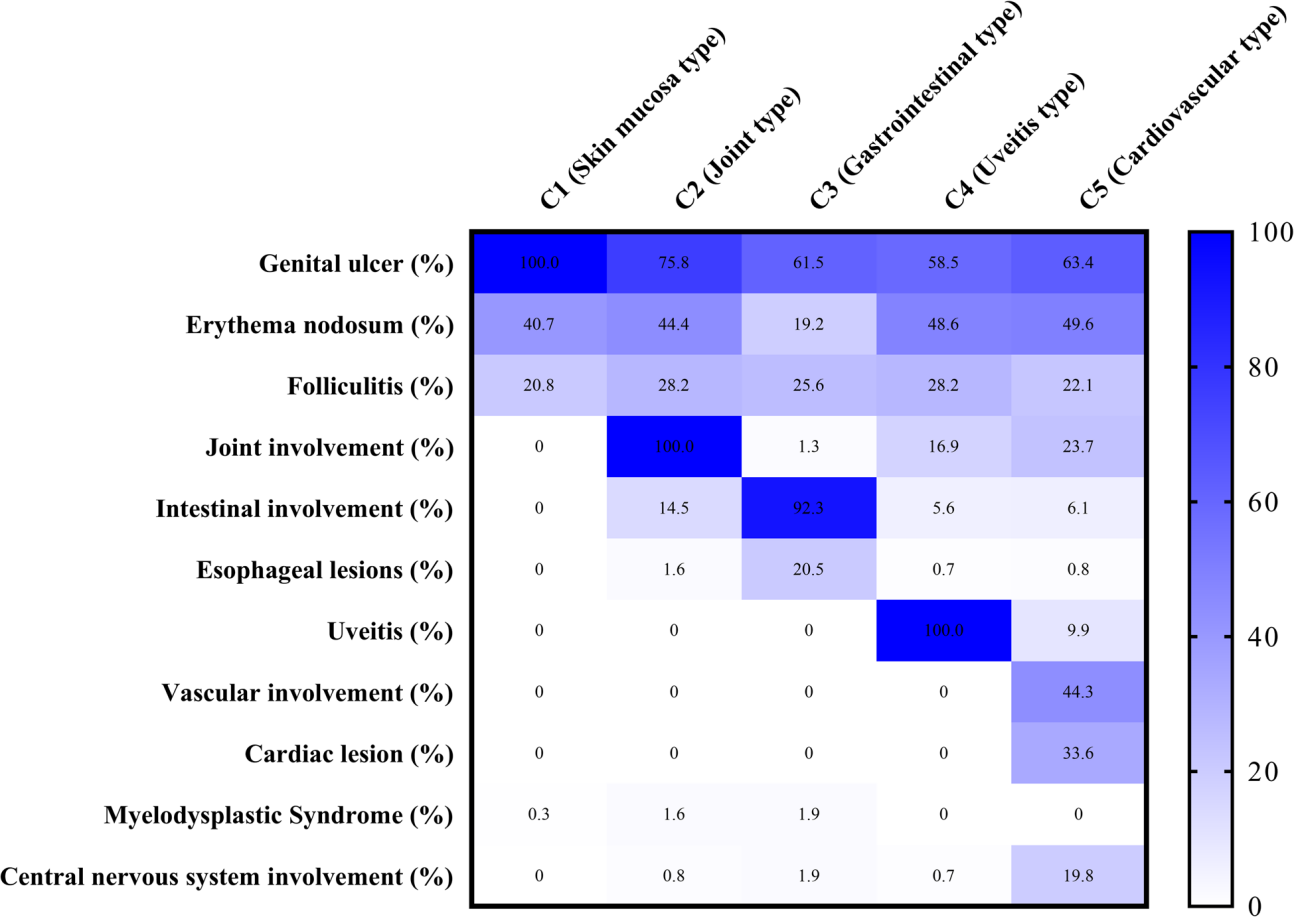
Five phenotype clusters of patients with Behçet’s syndrome

In C5 the median age at onset was 27 years (IQR 21–35 years), and 44.3% had vascular involvements. There were 29 cases with deep vein thrombosis and 7 cases with major vein (vena cava, hepatic) thrombosis and 46 cases with arterial thrombosis or aneurysms. Cardiac lesions were observed in 44 patients (40 having heart valvular disease, 2 having ventricular thrombi, 1 with coronary disease and the other with myocarditis). The majority of patients with CNS involvement were grouped in C5, including 15 cases with cerebral parenchymal lesions, 1 case with aseptic meningitis, 4 with CVST, 2 with arterial involvement, 3 cases with cerebral aneurysm and parenchymal lesions, and 1 with meningoencephalitic manifestations. Additionally, 13 cases had uveitis in C5. Vascular involvement was positively related to cardiac lesions [RR 2.40, (1.16, 4.96), *P* = 0.027] and CNS involvement [RR 4.41, (1.00, 9.22), *P* = 0.001]. Erythematous nodosum (58.6%) was more frequent in this cluster than in the others. 43.5%, 91.6%, 87.8%, and 43.5% subjects satisfied JPN, CHN, ICBD, and ISG criteria, respectively. The median Krause score was 6. Clinical characteristics of the five clusters of patients are summarized in Fig. 1.

## Discussion

We conducted detailed and comprehensive analyses in a large cohort of BS patients in China, from which we identified distinct clinical manifestations and major organ involvement patterns between male and female. As expected, major results in the present study were generally in line with those from previous reports [9, 10, 40]. We observed that male patients are characterized by papulopustular lesion-type skin lesions, ocular disease, cardiovascular disease, and central nervous system involvement, while females are characterized by high prevalence of genital ulcers.

The most striking sex effect we found was the links between male sex and cardiac, arterial diseases. As cardiac involvement and major vessel disease are the main cause of mortality in BS [41, 42], our study further supports the notion of the greater frequency of cardiac lesions and major vessel disease in men than in women. Cardiac lesions are rare manifestations of BS. The previous studies of sex-related phenotype either included too few cases to achieve statistical significance [9] or did not include cardiac and arterial disease for subgroup analysis [10]. On the contrary, we found that men were 2–5 times (more risk) as likely to have arterial disease and cardiac lesions as women.

Given the high diversity of clinical manifestations in BS, we applied cluster analysis to explore its phenotype patterns, which helped us to identify five distinct subgroups: C1, skin and mucosa type; C2, articular type; C3, GIBS type, the majority with intestinal involvements, and an aggregation of esophageal ulcers; C4, uveitis type, predominantly male with a younger age of onset; and C5, cardiovascular type and central nervous involvements. Each subset contains patients with only a small number of predominant clinical manifestations reflecting overall a low number of organs involved in our cohort.

Since BS is not a single disease, but a heterogeneous and multi-systemic complex syndrome, studies with well categorized BS subsets are essential [18]. A variety of combinations of clinical manifestations could link to different underlying pathologic pathways [43]. C1 contained most patients having only skin-mucosa lesions, which are the most common manifestations and could cause significant influence on quality of life [44], while in the other clusters, joint lesions and major organ involvements are responsible for serious morbidity and mortality [41] and mandate a variety of intensive treatment strategies.

To the best of our knowledge, no study has been published using cluster analysis of phenotype subsets in unrelated patients with BS. A recent study used cluster analysis to compare symptom patterns between familial and non-familial cases of BS [45]. It yielded a papulopustular lesions and arthritis cluster, which is similar to the results from a factor analysis study conducted by the same group [46]. This clinical feature is consistent with our findings in C2; subjects with articular involvement were associated with a higher prevalence of papulopustular lesions.

C3 consisted of the majority of the subjects having gastrointestinal involvements. Esophageal ulceration is an uncommon manifestation of BS [47]. Thirty-six cases with esophageal ulcers were observed and most of them were aggregated in C3. The other features of C3 were a low prevalence of ocular involvement and erythema nodosum, and no case of vascular involvement. The inverse association of intestinal involvements with ocular lesions was reported previously [8]. The absence of major manifestations (ocular involvement) and lower frequency of minor manifestations (erythema nodosum) resulted in an increment of possible BS cases in C3. Therefore, endoscopical findings are the most critical measurement for accurate diagnosis of intestinal BS [39]. Thus, all intestinal cases in our cohort were confirmed by distinctive endoscopic findings.

Intestinal ulceration is a clinical feature in BS associated with bone marrow failure (BMF), classified as conditions such as MDS or aplastic anemia, and associated with trisomy 8 [48, 49]. In line with those reports, the analysis of our cohort indicated a 4-fold greater risk of intestinal involvement for patients with MDS than without. Therefore, it is recommended for patients with MDS to undergo pretreatment colonoscopy evaluation.

A previous factor analysis study identified uveitis as a distinct factor, and it was negatively associated with erythema nodosum only among females [50]. In our cohort, we confirmed that uveitis was an entity by itself, prevailing in young male patients, rarely coexisting with intestinal involvements.

Of note, we identified cases with cardiovascular lesions grouped together in C5. Previous studies revealed a positive association between cardiac and vascular lesions [9, 51]. Cardiac involvements include myocarditis, aortic or mitral valve disease, intra-cavitary thrombi, and coronary damage. In our cohort, aortic or mitral valve disease was the most frequently involved damage to the heart.

By factor analysis, Krause et al. [52] identified a positive relation between deep vein thrombosis and neurologic involvement in BS. Similarly, we found patients with neurological involvement including parenchymal involvement and CVST were gathered in C5. The prevalence of neurological disorders is rare across race and ethnicity; the frequency of CVST is extremely low [53]. In a previous cohort, there were found 7/11 (64%) patients with CVST positively associated with extracranial large vessel events, compared with 15/77 (19%) patients with parenchymal disease (*p* = 0.004). Of note, in that cohort, there were only 11 cases with CVST, which could result in statistical bias. Due to ethnic differences and diverse statistical methods, we found the majority of neurological disorders were parenchymal lesions aggregated with cardiovascular lesions in C5. Besides, our results revealed that uveitis could cluster with vascular involvement [18].

Our findings could help us to identify clinical characteristics and understand the similarity of pathogenic mechanisms within each specific cluster, which could contribute to better ways of managing BS. With the current cluster pattern, we could presume a female with skin lesions would generally have a mild disease course, while a young male with panuveitis would be unlikely to have intestinal involvement. Cardiovascular and CNS involvements are clustered together, which suggests possible similar underlying pathogenesis in each manifestation. However, when applying this cluster pattern, we should consider phenotypic differences among racial and ethnic groups. As compared with cohorts from Middle East countries [54, 55], our cohort had a higher frequency of GI involvement and lower frequency of vascular and ocular lesions.

There are some strengths and limitations in this study. The major strength of our study was a well-defined large sample size and the strictly defined inclusion criteria which allowed a well-categorized the investigation of BS. Besides, we applied 18 variables to cluster analysis representing the disease’s heterogeneity. Additionally, we distinctly included data on intestinal and cardiac lesions, and diagnosis of MDS confirmed by objective laboratory findings. Nevertheless, a single-center study could lead to selection bias. It should be noted, in our cohort, that the prevalence of arterial lesion was higher than that of deep venous thrombosis. We cannot exclude a slight ascertainment bias for the prevalence of joint involvement. Finally, the cross-sectional design of our study and not including medication as a clustered variable did not allow us to investigate the dynamics of the phenotype. Therefore, a future, longitudinal study design is warranted for the stability of the cluster pattern.

## Conclusions

In conclusion, our data provided new insights into phenotype patterns in a large cohort of unrelated BS patients by a combination of sex-associated comparison and cluster analysis. Our preliminary findings of the subgroup pattern requires further replication to identify the similarity in other cohorts and even from other ethnicities. Whether the clustering solution can be translated into enhanced understanding of pathogenesis differences and guide therapy requires further clarification.

## Abbreviations

ABD: Adamantiades-Behçet’s disease
BMF: Bone marrow failure
BS: Behçet’s syndrome
CHN: The Chinese version of Behçet’s disease criteria created by Cheng and Zhang
CNS: Central neurological involvement
CT: Computed tomography
CVST: Cerebral venous sinus thrombosis
GIBS: Gastrointestinal Behçet’s syndrome
ICBD: International Criteria for BS
IQR: Interquartile range
ISG: International Study Group criteria
JPN: Diagnostic criteria of the Behçet’s disease research committee of Japan (1987 revision)
MDS: Myelodysplastic syndrome
MRI: Magnetic resonance imaging
RR: Relative risk
